# Implementation of an Evidence-Based Intervention with Safety Net Clinics to Improve Mammography Appointment Adherence Among Underserved Women

**DOI:** 10.1007/s13187-021-02116-w

**Published:** 2021-11-25

**Authors:** Jennifer Holcomb, Suja S. Rajan, Gayla M. Ferguson, Jiali Sun, Gretchen H. Walton, Linda Highfield

**Affiliations:** 1 Department of Management, Policy and Community Health, School of Public Health, The University of Texas Health Science Center at Houston (UTHealth), 1200 Pressler St, Houston, TX 77030 USA; 2 Sinai Urban Health Institute, 1500 South Fairfield Avenue, Chicago, IL 60608 USA; 3 Houston Hospice, 1905 Holcombe Blvd, Houston, TX 77030 USA; 4 Department of Epidemiology, Human Genetics and Environmental Sciences, School of Public Health, The University of Texas Health Science Center at Houston (UTHealth), 1200 Pressler St, Houston, TX 77030 USA; 5 Department of Internal Medicine, The University of Texas Health Science Center at Houston (UTHealth) John P and Katherine G McGovern Medical School, 6431 Fannin St, Houston, TX 77030 USA

**Keywords:** Evidence-based intervention, Mammography adherence, Implementation science, Consolidated Framework for Implementation Research, Safety net clinics

## Abstract

The Peace of Mind Program is an evidence-based intervention to improve mammography appointment adherence in underserved women. The aim of this study was to assess effectiveness of the intervention and implementation of the intervention in safety net clinics. The intervention was implemented through a non-randomized stepped wedge cluster hybrid study design with 19 Federally Qualified Health Centers and charity care clinics within the Greater Houston area. A multivariable generalized estimating equation logistic regression was conducted to examine mammography appointment adherence. A survey assessing Consolidated Framework for Implementation Research constructs was also conducted with clinic staff prior to adoption and eight weeks post implementation. One-sided *t*-tests were conducted to analyze mean score changes between the surveys. A total of 4402 women (baseline period = 2078; intervention period = 2324) were included in the final regression analysis. Women in the intervention period were more likely to attend or reschedule their mammography appointment (OR = 1.30; *p* < 0.01) than those in the baseline period receiving usual care. Women who completed the intervention were more likely to attend or reschedule their mammography appointment than those who did not complete the intervention (OR = 1.62; *p* < 0.01). The mammography appointment no-show rates for those in the baseline period, in the intervention period, and who completed the intervention were, respectively, 22%, 19%, and 15%. A total of 15 clinics prior to adoption and eight clinics completed the survey at 8 weeks post implementation A statistically significant mean score decrease was observed in Inner Setting and in two Inner Setting CFIR constructs, Culture–Effort, and Implementation Climate. While the intervention improved mammography appointment adherence, there are opportunities to further integrate Consolidated Framework for Implementation Research constructs. **Trial registration**: Clinical trials registration number: NCT02296177.

**Supplementary Information:**

The online version contains supplementary material available at 10.1007/s13187-021-02116-w.

## Background

Breast cancer remains the second leading cause of cancer related death among women in the USA [1]. Mammography screening appointment adherence can be a critical component to receiving an early-stage breast cancer diagnosis [2]. Missing one mammography screening increases a woman’s likelihood of developing a late-stage breast cancer [2–5]. Underserved women are at higher risk for late-stage diagnosis due to lower mammography screening rates, no-show appointments, and increased time between referral, diagnostic examination and treatment [6–10]. Understanding and addressing barriers to mammography appointment adherence in women who are not able to receive routine mammography screenings is the best way to reduce health disparities. Mammography screening interventions addressing both the structural (transportation and cost) and psychosocial (fear) barriers can promote appointment adherence in underserved communities [9, 10]. Patient navigation and counseling evidence-based interventions (EBIs) addressing these barriers have demonstrated success in increasing mammography appointment adherence [9–12]. Although mammography screening interventions might alter mammography screening behavior, to be successful, interventions should be adapted and tailored to meet community needs [10–12].

Dissemination and implementation (D&I) practitioners aiming to implement a tailored intervention to intercede across cost and access to care among underserved communities have also partnered with safety net healthcare systems and Federally Qualified Health Centers (FQHCs). FQHCs provide culturally competent primary care and preventative services in underserved communities [10]. The nature of FQHCs lends them direct access to underserved women in need of mammography screening services, but also creates barriers to EBI adoption and implementation [13, 14]. There is a gap in understanding of effective D&I methods in successful interventions, particularly those aimed at addressing mammography appointment barriers within safety net clinics [15]. The current study utilized a theoretically based, D&I strategy to facilitate the implementation of an EBI with FQHCs and charity care clinics and mammography mobile providers to improve mammography appointment adherence in underserved women. The Peace of Mind Program (PMP) intervention is an evidence-based mammography screening intervention based on the Transtheoretical Model of Change adapted from a research tested program from the National Cancer Institute’s Research Tested Intervention Programs (RTIPs) database [15, 16]. In this study, the PMP intervention was expanded to include implementation components geared toward supporting implementation in safety net clinics, and by doing so, aimed to address the research to practice gap [15, 16]. We hypothesized that the intervention would improve mammography appointment adherence compared to usual care. The study objectives were to test the effectiveness of the intervention in improving mammography appointment adherence and to assess implementation of the intervention.

## Methods

### Intervention

PMP is an active listening, tailored telephone reminder call intervention to counsel women through barriers to mammography screening appointment attendance [15, 16]. Each woman during the intervention period received up to three reminder call attempts for their scheduled mammography screening appointment at the safety net clinic. If the woman did not answer on the first attempt, two additional attempts were made to reach the woman. If the woman answered the phone call, but did not consent to participate in the study, she was reminded about her appointment in the usual care manner of each site. If she answered and consented to participate, a state certified Community Health Worker (CHW) who made the call assessed the woman’s confidence in attending their scheduled mammography appointment, counseled the woman through barriers to attending the appointment (e.g., transportation, childcare), and recorded the woman’s responses in an online interface program designed in RedCap. The results of each reminder phone call, whether the call was completed or a message left, and the woman’s resulting mammography appointment attendance or no-show status were also recorded.

To adopt the intervention, clinics must have been members of the Breast Health Collaborative of Texas (BHCTexas) within the Greater Houston service area. BHCTexas is a statewide member network of breast cancer survivors, advocates, health care professionals, and organizations providing mammography and other breast health services.

BHCTexas and research team worked with participating clinics to align goals for mobile mammography drives and facilitated relationships with mobile providers as needed to support PMP implementation. Training support and onsite role modeling from BHCTexas CHWs was provided throughout implementation to clinic CHWs delivering the intervention. In addition, participating clinics must (1) have had a designation as a FQHC by the Health Resources and Services Administration (HRSA) or be a charity clinic which provides free or reduced cost care to underserved populations in their service area, (2) serve women between the ages of 40 to 64 years old who were at or below 200% of the Federal Poverty Level for a family of four and who lacked health insurance, (3) engage in provision of mammography screening services at least six times per year (three in baseline and in intervention), and (4) women at the clinic must have been in need of mammography screening and be scheduled for an upcoming appointment. Patients must have completed a clinical breast exam prior to their scheduled appointment per mobile provider requirements. A full description of the intervention development, adoption, implementation, and stakeholder engagement components has been reported elsewhere [15–17].

### Study Design

A non-randomized stepped wedge cluster hybrid design was used to assign clinics into two non-concurrent implementation waves with two to three groups in each wave [15]. Variation across clinics existed in frequency of mammography drives, number of patients scheduled in each drive, number of staff available to participate in PMP, existing relationships with mammography mobile providers, and available funding and resources which resulted in differences in clinic readiness to start the intervention. Due to these differences, the randomized allocation of the clinics into waves and groups as previously described was not possible [15]. Clinics with lower levels of readiness were assigned to later groups to benefit from more time in the implementation strategies. Each clinic served as its own control during the baseline period and was required to have at least three mammography drives during both the baseline and intervention period. Since each clinic served as its own control, blinding was not possible.

To test effectiveness, the outcome measure was mammography appointment adherence. A patient’s adherence was categorized as “0” for a no-show or cancelled appointment and categorized as “1” for an attended or rescheduled appointment. The independent variables of interest were two dichotomous variables indicating study period, baseline period (categorized as “0”) or intervention period (categorized as “1”) and for the intervention period, whether the patient did not complete (categorized as “0”) or did complete (categorized as “1”) the intervention. A patient completed the intervention if they answered the reminder call, consented to the study, and received the staging question assessing confidence and barriers. Patient, intervention, clinic, and implementation covariates were also examined. Patient age was categorized into three age groups—55 and above, 45 to 54 and 25 to 44 years—to align with the age-based mammography screening guidelines. The season in which the patient scheduled their appointment was categorized by winter (January to March), spring (April to June), summer (July to September), and fall (October to December) to examine a possible seasonal effect [18, 19]. Wave was categorized as a dichotomous variable (0/1) for wave 1 and 2 and group was categorized as a three-category variable (1, 2, 3) based on when the clinic began the intervention. Each of the three mammography mobile providers were categorized as a dichotomous variable based on if the provider assisted with mammography screenings at the clinic in which the patient was scheduled (categorized as “1”) or not scheduled (categorized as “0”) for a mammography appointment. The provider with an existing reminder call and group education program for usual care was defined as the reference group. The clinic racial/ethnicity distribution (percentage) of the population served by each clinic from 2015 to 2016 was collected from the HRSA Uniform Data System for FQHCs and the Texas Association of Community Health Centers for charity care clinics. The clinics were categorized based on the racial/ethnicity group with the highest percentage in five mutually exclusive groups: non-Hispanic Black, non-Hispanic white, non-Hispanic other (another race other than Black or white), Hispanic, and multi-racial/ethnicity group (equal percentage of non-Hispanic Black, non-Hispanic other, and Hispanic women served). Each of the five groups were categorized as a dichotomous variable indicating if the racial/ethnicity group was the highest reported for the clinic (categorized as “1”) or not (categorized as “0”). The CHW who made the reminder call was a dichotomous variable based on if they were a clinic (categorized as “0”) or BHCTexas (categorized as “1”) staff member. A dichotomous variable determined if the patient answered the reminder call (categorized as “1”) or not (categorized as “0”). The number of reminder call attempts received by patient in the intervention period was a dichotomous variable for one call (categorized as “0”) or multiple call attempts (two or three calls categorized as “1”). Language was categorized by English, Spanish, or Vietnamese. To assess implementation, a survey was conducted with clinic leadership and staff with any potential role in PMP prior to adoption of the intervention and eight weeks into implementation to assess the same Consolidated Framework for Implementation Research (CFIR) constructs. A total of 75 survey statement items were used to assess twelve constructs across three CFIR domains using a survey adapted from the Cancer Prevention and Control Research Network for cancer control EBIs with FQHCs [Appendix] [20–22]. A mean score for each clinic was created to measure level of agreement with survey statement items (5 = completely agree to 1 = completely disagree). Twenty survey statements were recoded to align with level of agreement and scoring direction (e.g., *It will be hard to train providers and staff to implement the PMP*).

### Data Analysis

A descriptive analysis was performed to examine differences in covariates across the baseline and intervention periods. To test for statistically significant differences in mammography appointment adherence, we used chi-square tests. We used a multivariable generalized estimating equation (GEE) regression model to examine mammography appointment adherence in two analytical models. In the first model, we included all patients in the baseline and intervention period (intent to treat analysis). In the second model, we included only patients in the intervention period to examine those who did and did not complete the intervention (i.e., completed the reminder phone call). We modeled clustering across the 19 clinical sites using a logistic GEE regression (logit link with odds ratio) and an independent correlation structure. We analyzed age, season, wave, group, mammography mobile provider, the group variable and the five dichotomous variables for clinic racial/ethnic distribution, and for those in the intervention period, the CHW who made the appointment reminder call, if the patient answered the call, the number of reminder call attempts, and language independently in each of the models and added each additional variable as a covariate. The Quasi-Akaike information criterion (QIC) value was used to identity covariates to include in the final models. One-sided t-tests were conducted to analyze mean score changes (mean difference < 0) between the clinic adoption and implementation survey responses. All analyses were performed using Stata 14.0 (College Station, TX) with *α* = 0.05 as the limit for statistical significance.

## Results

**Fig. 1:**
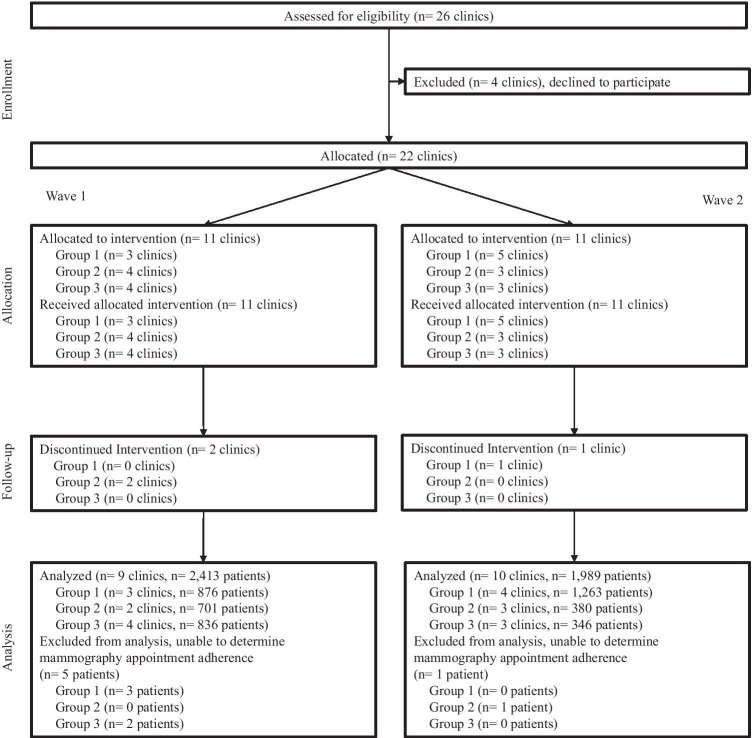
Flowchart showing enrollment, allocation, follow-up, and analysis of clinics and patients in the Peace of Mind Program (PMP) study trial. Description: Black and white graphic with a downward sequence of stages of enrollment, allocation, follow up, and analysis with indicated study participant sample size in each stage

Twenty-six FQHC and charity care clinical locations were approached for study enrollment (Fig. 1). Of the 26 clinics, 22 elected to adopt PMP (85%). Two clinics enrolled (three clinical locations), but did not complete the trial yielding a total of 19 clinical sites for analysis. A total of 4408 patients were recruited for the study. Six patients were excluded from the final analysis because we were unable to determine mammography appointment adherence outcome from the Redcap responses. Of the 4402 in the final analysis, 2078 were enrolled in the baseline period and 2324 were enrolled in the intervention period (Table 1). Women aged 45 to 54 years old made up the largest age group in both periods (baseline—45%; intervention—41%). The patients in the baseline intervention had a higher percentage of mammography attendance in the Summer (baseline—47%; intervention—18%) compared to those intervention period who had a higher attendance in the Spring (baseline—8%; intervention—36%). Over half of patients in both periods were in wave 1 (baseline—59%; intervention—51%). Group 3 was largest in the baseline intervention (baseline—41%; intervention—14%) while group 1 was the largest in the intervention period (baseline—26%; intervention—69%). Mammography provider 2 who had no existing reminder call or group education was the largest mammography mobile provider to women in both periods (baseline—48%; intervention—52%). Hispanic women were the largest served racial/ethnicity group across clinics in both periods (baseline—77%; intervention—70%).

**Table 1:** Descriptive statistics of patient, intervention, clinic, and implementation variables

	All Patients (*N* = 4402)		Intervention Period (*N* = 2324)	
*Variable*	Baseline Period (*n* = 2078)	Intervention Period (*n* = 2324)	Did not complete intervention (*n* = 752)	Completed intervention (*n* = 1572)
*Patient Variables*				
Age category (years)				
25 to 44	519 (25.0%)	549 (23.6%)	174 (23.1%)	375 (23.9%)
45 to 54	929 (44.7%)	958 (41.2%)	317 (42.2%)	641 (40.8%)
55 and older	630 (30.3%)	817 (35.2%)	261 (34.7%)	556 (35.4%)
*Intervention Variables*				
Season (months)				
Winter (Jan—March)	382 (18.4%)	537 (23.1%)	186 (24.7%)	351 (22.3%)
Spring (Apr—June)	172 (8.3%)	842 (36.2%)	256 (34.0%)	586 (37.3%)
Summer (July—Sep)	981 (47.2%)	414 (17.8%)	145 (19.3%)	269 (17.1%)
Fall (Oct—Dec)	543 (26.1%)	531 (22.9%)	165 (22.0%)	366 (23.3%)
Wave				
1	1233 (59.3%)	1180 (50.8%)	434 (57.7%)	746 (47.5%)
2	845 (40.7%)	1144 (49.2%)	318 (42.3%)	826 (52.5%)
Group				
1	543 (26.1%)	1596 (68.7%)	484 (64.4%)	1112 (70.7%)
2	676 (32.5%)	405 (17.4%)	169 (22.5%)	236 (15.0%)
3	859 (41.3%)	323 (13.9%)	99 (13.2%)	224 (14.3%)
*Clinic Variables*				
Mammography Mobile Provider				
Provider 1	919 (44.2%)	627 (27.0%)	220 (29.3%)	407 (25.9%
Provider 2	995 (47.9%)	1218 (52.4%)	403 (53.6%)	815 (51.8%)
Provider 3	164 (7.9%)	479 (20.6%)	129 (17.2%)	350 (22.3%)
Clinic racial distribution^a^				
Non-Hispanic Black	168 (8.1%)	119 (5.1%)	42 (5.6%)	77 (4.9%)
Non-Hispanic white	164 (7.9%)	415 (17.9%)	119 (15.8%)	296 (18.8%)
Non-Hispanic other	108 (5.2%)	145 (6.2%)	9 (1.2%)	136 (8.7%)
Hispanic	1591 (76.6%)	1627 (70.0%)	574 (76.3%)	1053 (67.0%)
Multi-racial/ethnicity group	47 (2.3%)	18 (0.77%)	8 (1.1%)	10 (0.64%)
*Implementation Variables*				
CHW calling patient	-	-		
Clinic staff	-	-	242 (32.2%)	405 (25.8%)
BHCT staff	-	-	510 (67.8%)	1167 (74.2%)
Answered reminder call	-	-		
No	-	-	465 (61.8%)	0 (0%)
Yes	-	-	287 (38.2%)	1572 (100%)
Number of reminder calls (*n* = 1852)^b^	-	-		
1 call	-	-	273 (45.7%)	908 (72.35%)
Multiple calls (2 or 3 calls)	-	-	324 (54.3%)	347 (27.65%)
Language of reminder call (*n* = 1817)^b^	-	-		
English	-	-	124 (50.6%)	608 (39.0%)
Spanish	-	-	121 (49.4%)	828 (53.0%)
Vietnamese	-	-	0 (0%)	125 (8.0%)

^a^Non-Hispanic Black, Non-Hispanic other, and Hispanic ^b^ Missing data

Of the 2324 patients in the intervention period, 1572 completed the intervention. Patient age group, season, and mammography mobile provider were similar across those who did and did not complete the intervention (Table 1). The percentage of patients who did not complete the intervention was higher in wave 1 (did not complete—58%; completed—47%) while 71% of those who completed the intervention were in group 1. With Hispanic women being the largest racial/ethnicity group, a higher percentage of those who did not complete the intervention were from a clinic serving predominately Hispanic women (did not complete—76%; completed—67%). A higher percentage of those who completed the intervention received a call from a BHCTexas CHW compared to a clinic CHW (did not complete—68%; completed—74%). For those who did not complete the intervention, 38% answered the reminder call. For those who completed the intervention, 72% received one reminder phone call compared to 46% of those who did not complete the intervention. A higher percentage of patients who did not complete the intervention received a call in English (did not complete—51%; completed—39%) while a higher percentage of those who completed the intervention received the call in Spanish (did not complete—49%; completed—53%). All patients who received a call in Vietnamese completed the intervention.

### Mammography Appointment Adherence

In the bivariate analysis, multiple statistically significant differences in mammography appointment adherence were identified (Table 2). A statistically significant difference in appointment adherence was observed between periods (*p* < 0.05), completion of the intervention (*p* < 0.001), wave (*p* < 0.01), group (*p* < 0.001), mammography mobile provider (*p* < 0.001), and clinic racial distribution (*p* < 0.001). A marginal trend toward significance (*p* = 0.058) was observed for both age and season. Those who attended or rescheduled their mammography appointment were more likely to be in the intervention period, complete the intervention, be in wave 1 and group 1, be served by a clinic serving predominately non-Hispanic women who identified with a race other than Black or white, and be served by mammography mobile provider 1 who had an existing reminder call and group education program compared to those who did not show or cancelled their appointment. The number of reminders call attempts, if the patient answered the reminder call, and language of the reminder call were statistically significant (*p* < 0.001). Those who attended or rescheduled their mammography appointment were more likely to answer the reminder call, receive one reminder call, and receive a reminder call in Spanish or Vietnamese compared to those who did not show or cancelled their appointment. Among the 2078 patients in the baseline period, 448 (22% no-show rate) did not show up to their appointment, whereas among the 2324 patients in the intervention period 438 (19% no-show rate) did not show up to their appointment. Among the 752 patients who did not complete intervention 205 (27% no-show rate) did not show up, whereas among the 1572 patients who completed the intervention 233 (15% no-show rate) did not show up. Table 3 includes the multivariable GEE logistic regression performed to fit the covariates to mammography appointment adherence by baseline and intervention period and completion of the intervention. In the first model, the intervention period, relative to the baseline period, was associated with higher odds of attending or rescheduling a mammography appointment (OR = 1.30; *p* < 0.01). The age group of 25 to 44 years was associated with lower odds compared to the 55 years and older age group (OR = 0.73; *p* < 0.001). Mammography mobile provider 2 was associated with lower odds (OR = 0.60; *p* < 0.01) compared to mammography mobile provider 1. In the second model, completing the intervention, relative to not completing, was associated with higher odds of attending or rescheduling an appointment (OR = 1.62; *p* < 0.01). As in the first model, the age group of 25 to 44 years was associated with lower odds compared to the 55 years and older age group (OR = 0.71; *p* < 0.05). Relative to receiving one reminder call, receiving multiple reminder call attempts was also associated with lower odds attending or rescheduling an appointment (OR = 0.78; *p* < 0.05).

**Table 2:** Association of patient, intervention, clinic, and implementation variables with mammography appointment adherence (*N* = 4402)

*Variable*	No Show or Cancelled (*N* = 886)	Attended or Rescheduled (*N* = 3516)	*p* value
Study period			< 0.05
Baseline period	448 (50.6%)	1630 (46.5%)	
Intervention period	438 (49.4%)	1886 (53.6%)	
Intervention period (n = 2324)			< 0.001
Did not complete intervention	205 (46.8%)	547 (29.0%)	
Completed intervention	233 (53.2%)	1339 (71.0%)	
*Patient Variables*			
Age category (years)			NS
25 to 44	239 (27.0%)	829 (23.6%)	
45 to 54	379 (42.8%)	1508 (42.9%)	
55 and older	268 (30.3%)	1179 (33.5%)	
*Intervention Variables*			
Season (months)			NS
Winter (Jan—March)	178 (20.1%)	741 (21.1%)	
Spring (Apr—June)	202 (22.8%)	812 (23.1%)	
Summer (July—Sep)	299 (33.8%)	1096 (31.2%)	
Fall (Oct—Dec)	207 (23.4%)	867 (24.7%)	
Wave			< 0.01
1	450 (50.8%)	1963 (55.8%)	
2	436 (49.2%)	1553 (44.2%)	
Group			< 0.001
1	396 (44.7%)	1743 (49.6%)	
2	201 (22.7%)	880 (25.0%)	
3	289 (32.6%)	893 (25.4%)	
*Clinic Variables*			
Mammography Mobile Provider			
Provider 1	252 (28.4%)	1294 (36.8%)	< 0.001
Provider 2	511 (57.7%)	1702 (48.4%)	
Provider 3	123 (13.9%)	520 (14.8%)	
Clinic racial distribution			< 0.001
Non-Hispanic Black	97 (11.0%)	190 (5.4%)	
Non-Hispanic white	121(13.7%)	480 (13.0%)	
Non-Hispanic other	12 (1.4%)	241 (6.9%)	
Hispanic	650 (73.4%)	2568 (73.0%)	
Multi-racial/ethnicity^a^	6 (0.68%)	59 (1.7%)	
*Implementation Variables*			
CHW calling patient (*n* = 2324)			NS
Clinic staff	114 (26.0%)	533 (28.3%)	
BHCT staff	324 (74.0%)	1353 (71.7%)	
Answered reminder call (*n* = 2324)			< 0.001
No	139 (31.7%)	326 (17.3%)	
Yes	299 (68.3%)	1560 (82.7%)	
Number of reminder calls (*n* = 1852)^b^			< 0.001
1 call	202 (54.0%)	979 (66.2%)	
Multiple calls (2 or 3 calls)	172 (46.0%)	499 (33.8%)	
Language of reminder call (*n* = 1806)^b^			< 0.001
English	148 (52.5%)	584 (38.3%)	
Spanish	132 (46.8%)	817 (53.6%)	
Vietnamese	2 (0.71%)	123 (8.1%)	

^a^Non-Hispanic Black, Non-Hispanic other, and Hispanic ^b^ Missing data

**Table 3:** Generalized estimating equation (GEE) models assessing mammography appointment adherence by baseline and intervention period and completion of the intervention

	Model 1 (*N* = 4402)		Model 2 (*N* = 1852)	
*Variable*	*Odds Ratio*	*SE*	*Odds Ratio*	*SE*
Study period	1.30**	0.12	-	-
Intervention period^a^	-	-	1.62**	0.30
Age category (years)	-	-		
25 to 44 vs 55 and older	0.73***	0.06	0.71*	0.12
45 to 54 vs 55 and older	0.87	0.09	1.04	0.19
Season (months)				
Spring vs Winter	0.95	0.13	0.98	0.22
Summer vs Winter	0.95	0.14	0.84	0.25
Fall vs Winter	1.03	0.16	1.16	0.33
Wave	0.95	0.25	0.98	0.28
Mobile Mammography Provider 2	0.60**	0.11	0.77	0.16
Mobile Mammography Provider 3	0.63	0.2	1.09	0.35
Clinic racial distribution—Hispanic	0.82	0.37	1.52	0.58
CHW calling patient	0.96	0.12	0.87	0.12
Answered reminder call	-	-	1.41	0.32
Number of reminder calls	-	-	0.78*	0.09

^a^ Did not or did complete the intervention

* *p* < .05 ** *p* < .01 *** *p* < .001

### Implementation

A total of 20 clinics completed the adoption survey prior to implementation, with 15 of these clinics adopting and implementing the intervention. Of those 15 clinics who completed the adoption survey and implemented the intervention, eight completed the implementation survey at eight weeks post implementation. While we observed a statistically significant (*p* < 0.05) decreases in Inner Setting overall and in Culture-Effort and Implementation Climate (Inner Setting constructs), potential directional trends can be identified. We observed a decrease in intervention Characteristic constructs such as Relative Advantage, Trialability, and Compatibility, but an increase in Complexity (i.e., easier to implement). We observed an increase in the Inner Setting construct of Culture–Stress (i.e., improvements in staff stress and frustration) and in the Outer Setting constructs of Policies and Incentives and Patients Needs and Resources. The implementation survey included questions to assess motivation to participate, PMP enrollment, and influence of the adoption webinar. Across the eight clinics, a total of 16 clinic staff members completed these questions. Clinics staff members reported their motivation to participate in PMP included participating in an EBI, helping patients to understand the importance of mammography screening, and reducing mammography no-show rates. Staff members reported being more motivated to participate in PMP because of the partnership between BHCTexas and the researchers, compared to just their membership in BHCTexas. All but one clinic staff member who participated in the webinar reported the webinar influenced their decision to enroll in PMP. All clinic staff members found the enrollment for PMP easy.

## Discussion

Within the cancer realm, a wide gulf between research and practice continues to lead to suboptimal EBI implementation [23]. We sought specifically to address these gaps in the development of PMP and in partnering with BHCTexas in this study [15, 16]. We hypothesized that a community-academic partnership would positively impact adoption and implementation. Bridging factors in implementation science consider relational ties, strength, processes and formal arrangements that connect the Inner and Outer Setting [24]. Recent research indicates a gap in the implementation science literature related to bridging and its impact on EBI implementation [24]. Using a community-academic partnership, focusing on internal and external incentives, addressing funding gaps, and staging implementation were all applied in the implementation strategies [15, 16]. The implementation survey indicated this partnership motivated clinics to adopt. We found a directional (though not significant) increases in Outer setting constructs related to bridging (Patient Needs and Resources) and in Inner Setting constructs related to ease of implementation (Culture–Stress). We also found statistically significant decreases in some Inner Setting constructs (Culture-Effort and Implementation Climate). It is possible that 8 weeks post-implementation was too soon for staff to feel that sufficient systems to support PMP were in place in their clinics and that they had sufficient self-efficacy to lead without support. It also is possible that Inner Setting scores decreased at implementation due to staff gaining a more realistic understanding of what successful EBI implementation takes once being exposed to PMP in daily operations. This change could reflect the dynamic nature of working in under-resourced, high stress clinical environments. Our previous work in PMP development and results from this study provide an opportunity to expand the knowledge base related to bridging strategies on EBI implementation, highlight practical approaches that can be replicated or built upon by other implementation scientists and identifies opportunities where further study is warranted (such as the effect on Inner Setting constructs). No differences in attendance were observed by race in the intervention period model indicating that PMP addressed barriers across diverse population groups. Further, language in which PMP was delivered, which was statistically significant in bivariate analysis, was not significant in the intervention period model. This indicates that PMP delivery was effective in multiple languages. Marginal trends observed for season disappeared in the intervention period model indicating PMP successfully addressed structural barriers that have been shown to affect patient appointment attendance during the year (such as lack of time off). Effect of mobile provider’s usual care reminder and education practices on attendance also disappeared in the GEE model for women who completed our program, indicating that PMP successfully improved appointment adherence to an equivalent level across providers despite this variation in routine care delivery.

Limitations of the study include not randomizing the start dates of clinics, which could have resulted in bias. We evaluated the effect of wave and group in our GEE models and found no impact. Women of younger age did retain lower odds of attendance even when completing PMP, indicating that there may be unique barriers in this age group. This finding should be investigated further to understand if younger women face unique barriers to adherence. We also conducted the implementation survey at eight weeks post implementation for each site. There is no literature to indicate the ideal timing for assessment of these constructs after implementation. The adoption and implementation survey were provided to all clinic staff, though not all sites completed them despite reminders. Due to low numbers of staff completing both surveys, we were not able to perform more advanced statistical analysis. Due to the low response rate, the external generalizability of the findings should be considered, as the staff or clinics completing the survey could have had differences in motivation or readiness to implement compared to those who did not complete the survey. High rates of staff turnover did occur in the clinics during the study which required ongoing training and support and may impact sustainability of the implementation strategies (e.g., trainings, stakeholder meetings, support) and the intervention.

## Conclusion

The findings address a research to practice gap in understanding effective implementation of a mammography EBI. Identifying clinic readiness for implementation and providing implementation strategies to support clinics are important to promote the successful uptake of an EBI in safety net clinics.

## Supplementary Information

Below is the link to the electronic supplementary material.

Supplementary file1 (PDF 161 kb)

## Data Availability

The datasets generated and/or analyzed during the current study are not publicly available due identifying clinic and patient information but are available from the corresponding author on reasonable request.
