# Supplementary material for: Implementation of an Evidence-Based Intervention with Safety Net Clinics to Improve Mammography Appointment Adherence Among Underserved Women
Source: J Cancer Educ. 2021 Nov 25;38(1):309–18. doi: 10.1007/s13187-021-02116-w (PMC9852109; doi:10.1007/s13187-021-02116-w)
Supplement: Supplementary file 1 — Supplementary file1 (PDF 161 kb) [file 13187_2021_2116_MOESM1_ESM.pdf]

<u>Peace of Mind Program (PMP) Adoption and Implementation Survey</u>

Mammography Intervention Characteristics (Intervention Characteristics Domain)
Relative advantage

1. Using BHC Peace of Mind Program (PMP) will be more effective than our prior practices for increasing mammography appointment attendance rates
2. Using BHC Peace of Mind Program will provide an important benefit to our patients

Complexity

3. It will be hard to train providers and staff to implement the PMP
4. It will be hard to train someone on the staff to do the PMP telephone counseling
5. It seems like it will be pretty easy to keep the PMP program going
6. It will be complicated to implement PMP
7. Using the PMP will require our clinic to make substantial changes to our way of doing things
8. Using the PMP will require more work than our current funding will support

Trialability

9. Once we try the PMP it will not be easy to go back to our old way of doing things even if we do not like it
10. To try the PMP we will have to make systems changes that will be permanent
11. If we try the PMP and don't like it, it will be pretty easy to change it or go back to our old practices

Compatibility

12. Using the PMP to increase mammography appointment attendance rates is compatible with current activities / practices in the clinic
13. Using the PMP to increase mammography fits well with the way I like to work
14. Using the PMP to increase mammography fits well with our current goals for women's health
15. The PMP fits well with the way our providers like to do things
16. Using BHC Peace of Mind Program fits with our high priority to improve our breast health services

Organization – Internal Environment (Outer Setting Domain)
Policies and Incentives

17. Our health center is required to report mammography rates to an outside organization / funder?
18. Mammography rate is an important quality metric for our health center
19. During the past fiscal year, our health center received [additional] income / reimbursement attached to mammography rates or associated quality metrics
20. During the past fiscal year, our health center was recognized or rewarded for doing a good job with mammography (for example, public recognition; professional association recognition)

Organization – Internal Environment (Inner Setting Domain)
Readiness for Change / Change Processes

21. The clinic leadership will make sure that we have the time to implement the PMP
22. The clinic leadership will make sure that we have the systems in place to implement the PMP
23. Clinic leadership creates an environment in which new programs like the PMP can be accomplished
24. Someone at our clinic usually steps up to provide leadership to implement new programs (becomes a program champion to actively support and promote new programs)
25. Some of our staff (i.e., managers, supervisors, other staff), will become program champions, actively supporting and promoting the PMP beyond what is required.

26. Clinic staff takes an active interest in programmatic-related problems and successes.
27. Managers will actively support implementation of the PMP
28. Our clinic needs help to implement the PMP
29. Our clinic needs help to implement other evidence-based programs

Patient Needs and Resources **(Outer Setting Domain)**
30. This clinic does a good job of assessing patient needs and expectations
31. Clinic staff promptly resolves patient complaints.
32. Patients' complaints are studied to identify patterns and prevent the same problems from recurring.
33. This clinic uses data on patient expectations and/or satisfaction when developing new services
34. This clinic uses data from patients to improve care.
35. This clinic does a good job of assessing patient needs and expectations regarding women's health
36. This clinic does a good job of assessing breast health needs and expectations
37. Our clinic needs to improve breast health services to better meet the needs of patients
38. Our clinic needs to increase access to mammography to better meet the needs of patients
39. Our clinic needs to help patients become compliant with mammography screening recommendations

Culture (General)
40. At our clinic, people openly talk about what is and isn't working
41. Most people in this clinic are willing to change how they do things in response to feedback from others.
42. After trying something new, we take time to think about how it worked
43. Most of the people who work in our clinic seem to enjoy their work
44. It is hard to get things to change in our clinic
45. Difficult problems are solved through face-to-face discussions.
46. People in this clinic operate as a real team
47. I can rely on the other people in this clinic to do their jobs well.
48. We regularly take time to reflect on how we do things.
49. Our clinic needs help to better communicate openly about what is and isn't working.
50. Our clinic staff needs help to better communicate openly about what is and isn't working regarding mammography services.
51. Our clinic staff needs help to work together to improve quality of mammography services.
52. Our clinic staff needs help to work together to implement
53. Most people in this clinic are willing to change how they do things
54. Throughout the clinic there is frequent and good communication about how different changes are going.
55. We use data to guide our clinic (e.g., performance reviews, assessments).

Culture stress
56. I am under too many pressures to do my job effectively
57. Our clinic staff members often show signs of stress and strain
58. The heavy workload in our clinic reduces our ability to successfully implement a program like PMP
59. Staff frustration is common in our clinic

Culture effort
60. People in our clinic always want to perform to the best of their abilities
61. People in our clinic are enthusiastic about their work

62. People in our clinic are enthusiastic about the PMP program
63. People in our clinic get by with doing as little as possible
64. People in our clinic do no put more effort into their work than they have to

Implementation climate

65. Clinic staff are expected to help PMP meet its goal (i.e., increase mammography appointment attendance rates)
66. Clinic staff will get the support they need to implement PMP
67. Clinic staff will get recognition for implementing PMP to increase mammography appointment attendance rates
68. Using PMP to increase mammography appointment attendance rates is a top priority for our clinic

Available Resources

69. In general, when there is agreement that change needs to happen in the clinic we have the necessary budget or financial resources
70. In general, when there is agreement that change needs to happen in the clinic we have the necessary training
71. In general, when there is agreement that change needs to happen in the clinic we have the necessary staffing
72. There are the equipment and materials to support the PMP in our clinic
73. There is enough patient awareness and need to support the PMP in our clinic
74. There is provider buy-in to support the PMP in our clinic
75. There is an implementation team to support the PMP in our clinic
